# Propionate Production from Carbon Monoxide by Synthetic Cocultures of Acetobacterium wieringae and Propionigenic Bacteria

**DOI:** 10.1128/AEM.02839-20

**Published:** 2021-06-25

**Authors:** João P. C. Moreira, Martijn Diender, Ana L. Arantes, Sjef Boeren, Alfons J. M. Stams, M. Madalena Alves, Joana I. Alves, Diana Z. Sousa

**Affiliations:** a Centre of Biological Engineering, University of Minho, Braga, Portugal; b Laboratory of Microbiology, Wageningen University & Research, Wageningen, The Netherlands; c Laboratory of Biochemistry, Wageningen University & Research, Wageningen, The Netherlands; University of Michigan—Ann Arbor

**Keywords:** syngas, carbon cycling, gas fermentation, acetogen, cocultivation, microbial interactions

## Abstract

Gas fermentation is a promising way to convert CO-rich gases to chemicals. We studied the use of synthetic cocultures composed of carboxydotrophic and propionigenic bacteria to convert CO to propionate. So far, isolated carboxydotrophs cannot directly ferment CO to propionate, and therefore, this cocultivation approach was investigated. Four distinct synthetic cocultures were constructed, consisting of Acetobacterium wieringae (DSM 1911^T^) and Pelobacter propionicus (DSM 2379^T^), *Ac. wieringae* (DSM 1911^T^) and Anaerotignum neopropionicum (DSM 3847^T^), *Ac. wieringae* strain JM and *P. propionicus* (DSM 2379^T^), and *Ac. wieringae* strain JM and *An. neopropionicum* (DSM 3847^T^). Propionate was produced by all the cocultures, with the highest titer (∼24 mM) being measured in the coculture composed of *Ac. wieringae* strain JM and *An. neopropionicum*, which also produced isovalerate (∼4 mM), butyrate (∼1 mM), and isobutyrate (0.3 mM). This coculture was further studied using proteogenomics. As expected, enzymes involved in the Wood-Ljungdahl pathway in *Ac. wieringae* strain JM, which are responsible for the conversion of CO to ethanol and acetate, were detected; the proteome of *An. neopropionicum* confirmed the conversion of ethanol to propionate via the acrylate pathway. In addition, proteins related to amino acid metabolism and stress response were highly abundant during cocultivation, which raises the hypothesis that amino acids are exchanged by the two microorganisms, accompanied by isovalerate and isobutyrate production. This highlights the importance of explicitly looking at fortuitous microbial interactions during cocultivation to fully understand coculture behavior.

**IMPORTANCE** Syngas fermentation has great potential for the sustainable production of chemicals from wastes (via prior gasification) and flue gases containing CO/CO_2_. Research efforts need to be directed toward expanding the product portfolio of gas fermentation, which is currently limited to mainly acetate and ethanol. This study provides the basis for a microbial process to produce propionate from CO using synthetic cocultures composed of acetogenic and propionigenic bacteria and elucidates the metabolic pathways involved. Furthermore, based on proteomics results, we hypothesize that the two bacterial species engage in an interaction that results in amino acid exchange, which subsequently promotes isovalerate and isobutyrate production. These findings provide a new understanding of gas fermentation and a coculturing strategy for expanding the product spectrum of microbial conversion of CO/CO_2_.

## INTRODUCTION

With the increasing interest in circular bioeconomy, acetogens have gained attention for their ability to produce commodities from one-carbon compounds, such as CO, CO_2_ (+H_2_), or formate (1–4). In particular, CO is an excellent electron donor for acetogens because of its low reduction potential (E_0_′ [CO_2_/CO] ∼ −520 mV) (5). With CO, acetogens show a higher production of reduced products, such as ethanol, acetone, or butyrate, than when grown on H_2_ plus CO_2_ or formate (3, 4). Acetogens are already being applied by companies like Lanzatech to convert steel mill flue gases (rich in CO) to ethanol (6). Such applications could be expanded to other more sustainable CO sources. For example, CO-containing syngas can be obtained by gasification of lignocellulosic materials (7). More recently, the production of CO by electrochemical reduction of CO_2_ with high Faraday efficiencies (>80%) has also been shown (8–10).

Acetogens use the Wood-Ljungdahl pathway to convert CO to acetyl coenzyme A (acetyl-CoA), which can be assimilated into cell biomass or converted to acetate for ATP production; acetyl-CoA can also be reduced to, e.g., ethanol to regulate intracellular redox balance (4). Most acetogens produce acetate and ethanol from CO, with some strains also forming small amounts of butyrate, butanol, and 2,3-butanediol (6). Metabolic engineering of acetogens is creating new avenues for CO conversion; for example, Clostridium ljungdahlii has been modified to produce acetone and isopropanol (2, 11). However, because growth on CO/syngas is energy constrained, only products that do not require much ATP for synthesis can be effectively targeted using such approaches. An alternative to expand the product repertoire of CO/syngas fermentation is sequential cultivation or cocultivation of selected microorganisms. For example, sequential cultivation of *C. ljungdahlii* and Aspergillus oryzae was used to convert syngas to malic acid (12), and a coculture of Clostridium autoethanogenum and Clostridium kluyveri produced butyrate, caproate, butanol, and hexanol (13, 14).

Previously, we described an enrichment culture that produced acetate and propionate from CO (15). This culture was predominantly composed of microorganisms affiliated with Acetobacterium wieringae (87% relative abundance), and propionigenic bacteria closely related to Pelobacter propionicus and Anaerotignum neopropionicum (1 and 2%, respectively) (15). *Ac. wieringae* strain JM was subsequently isolated from this enrichment (15). When grown solely on CO, strain JM produced acetate and ethanol but not propionate. We theorized that acetogens and propionigenic bacteria were cross-feeding in the enrichment culture: acetogens converted CO into ethanol, and the propionigenic bacteria used ethanol to produce propionate. Propionate has several industrial applications, e.g., as an antifungal agent in food and feed and as a building block to produce plastics and herbicides (16). Its biological production from C_1_ compounds is reported only in open mixed cultures, where a mixture of volatile fatty acids is obtained (15). Approaches for microbial selective propionate production commonly involve *Propionibacterium* species, which can use sugars, glycerol, and lactate as substrates (16) but not CO or products from CO fermentation. Propionigenic bacteria such as *An. neopropionicum*, *P. propionicus*, and Desulfobulbus propionicus can produce propionate from the products of CO fermentation, such as from ethanol, 2,3-butanediol, and propanol plus acetate (17–20), and could therefore be coupled to gas-fermenting processes for the production of propionate.

Here, we show that *Ac. wieringae* can be cocultivated with propionigenic bacteria (*P. propionicus* or *An. neopropionicum*) producing propionate from CO. The physiology of CO conversion to propionate by the coculture composed of *Ac. wieringae* strain JM and *An. neopropionicum* was also assessed, including the effect of acetate amendment, the metabolic pathways, and interspecies microbial interactions.

## RESULTS

### CO conversion by synthetic cocultures of *Ac. wieringae* and propionigenic bacteria.

Cultures of *Ac. wieringae* (DSM 1911^T^), *Ac. wieringae* strain JM, *P. propionicus* (DSM 2379^T^), and *An. neopropionicum* (DSM 3847^T^) were grown in pure culture prior to the establishment of the cocultures; pure cultures of *Ac. wieringae* and strain JM were grown on CO, while pure cultures of *P. propionicus* and *An. neopropionicum* were grown on ethanol. Exponentially growing cultures of acetogens and propionigenic bacteria were added in a 1:1 (vol/vol) ratio to form four distinct cocultures: *Ac. wieringae* and *An. neopropionicum* (*Aw*-*An*), *Ac. wieringae* and *P. propionicus* (*Aw*-*Pp*), *Ac. wieringae* strain JM and *An. neopropionicum* (*JM*-*An*); and *Ac. wieringae* strain JM and *P. propionicus* (*JM*-*Pp*). These cocultures were further grown on CO as sole carbon and energy source (Fig. S1). Cocultures containing the *Ac. wieringae* type strain (*Aw*-*Pp* and *Aw*-*An*) consumed 82 and 57 mmol liter^−1^ of CO in 8 days, respectively (Fig. S1), and produced 4.6 and 13.0 mM propionate (Fig. S1). Cocultures with *Ac. wieringae* strain JM (*JM*-*An* and *JM*-*Pp*) showed higher CO consumption rates (111 and 103 mmol liter^−1^ in 5 days) (Fig. S1), but no propionate formation was observed during the 5 days of incubation.

**FIG 1:**
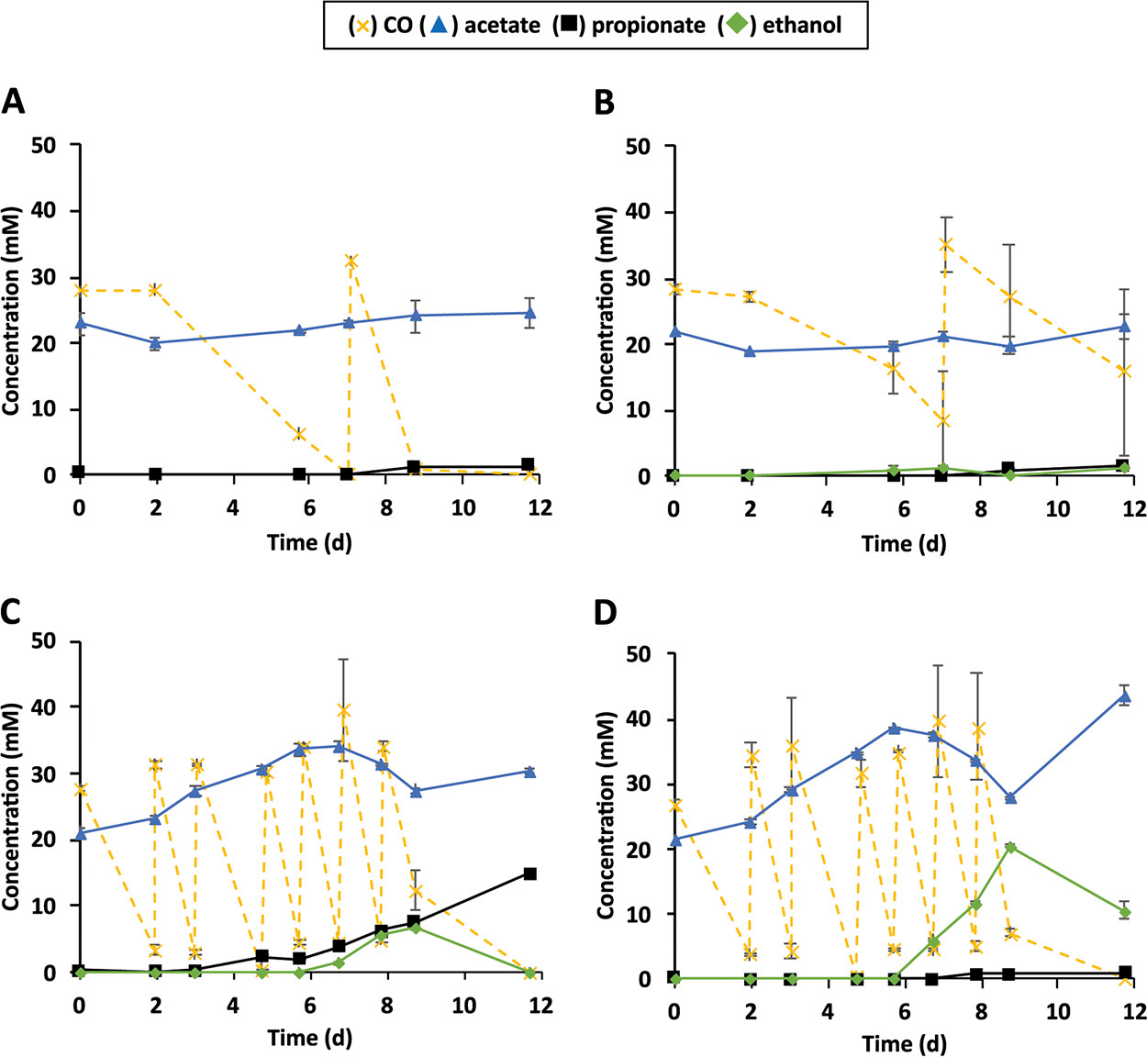
Growth of cocultures after the first transfer into a CO-acetate environment (CO-N_2_-CO_2_, 50%:30%:20% [vol/vol], 20 mM acetate). (A) *Ac. wieringae* plus *An. neopropionicum* (*Aw*-*An*). (B) *Ac. wieringae* plus *P. propionicus* (*Aw*-*Pp*). (C) *Ac. wieringae* strain JM plus *An. neopropionicum* (*JM*-*An*). (D) *Ac. wieringae* strain JM plus *P. propionicus* (*JM*-*Pp*). Error bars represent the standard deviations for biological duplicates (where error bars are not visible, the symbol is greater than the standard deviation).

All the first-generation cocultures (*Aw*-*An*, *Aw*-*Pp*, *JM*-*An*, and *JM*-*Pp*) were transferred to fresh medium (2% inoculum) with CO, but now including acetate (20 mM) (Fig. 1). Acetate addition was done to mimic the conditions of the propionate-producing enrichment from which strain JM was isolated (15). This resulted in propionate production by all four cocultures (Fig. 1). The final concentrations of propionate (12 days incubation) varied from 0.7 to 14.7 mM (Fig. 1); the coculture *JM*-*An* had the highest production of propionate (Fig. 1C). Final CO conversion in cocultures with strain JM was higher than in cocultures with *Ac. wieringae* type strain (Fig. 1): i.e., after 12 days of incubation, cocultures *JM*-*An* and *JM*-*Pp* converted about 200 mmol liter^−1^ CO, whereas *Aw*-*An* and *Aw*-*Pp* converted less than 60 mmol liter^−1^ CO (Fig. 1). In addition, propionate yield was higher in *JM*-*An* than in cocultures with *Ac. wieringae* type strain (Table 1). The coculture *JM*-*Pp* had the lowest propionate production (Fig. 1D), and ethanol accumulated (10.5 mM), indicating that the propionigenic strain was not very active.

**TABLE 1:** Overview of the fermentation profiles for different experiments with different cocultures^*a*^

Growth condition (acetate concn [mM])	Coculture	CO consumption (mmol liter^−1^)^*b*^	CO consumption rate (mmol liter^−1^ h^−1^)^*b*^^,^^*c*^	Propionate yield (mol propionate/mol CO)	Concn (mM) of product					
					Acetate^*d*^	Propionate	Ethanol	Isovalerate	Isobutyrate	Butyrate
First transfer of cocultures (20)	*Aw*-*An* (Fig. 1A)	58^*e*^	0.14^*e*^	0.019^*e*^	−2.1–5.7	1.1–1.2	0.0	ND	ND	ND
	*Aw*-*Pp* (Fig. 1B)	30–49	0.11–0.17	0.033–0.040	−1.2–2.8	1.2–1.6	0.9–2.8	ND	ND	ND
	*JM*-*An* (Fig. 1C)	180–194	0.64–0.69	0.075–0.083	9.0–9.3	14.5–14.9	0.0	ND	ND	ND
	*JM*-*Pp* (Fig. 1D)	190–202	0.68–0.72	0.0037–0.0038	20.5–23.6	0.7–0.8	9.3–11.8	ND	ND	ND

After subsequent transfers of cocultures (20)	*JM*-*An* (Fig. 2A)	231–257	0.64–0.75	0.081–0.113	−1.8–9.2	21.7–26.0	0.0	2.6–2.9	0.2–0.4	0.4–0.7

After subsequent transfers of cocultures (30)	*JM*-*An* (Fig. 2B)	194–221	0.82–0.94	0.080–0.140	−19.5 to −21.9	17.7–27.2	0.0	3.4–4.1	0.1–0.2	0.7–1.1

a *Aw*-*An*, *Ac. wieringae* plus *An. neopropionicum*; *Aw*-*Pp*, *Ac. wieringae* plus *P. propionicus*; *JM*-*An*, *Ac. wieringae* strain JM plus *An. neopropionicum*; *JM*-*Pp*, *Ac. wieringae* strain JM plus *P. propionicus*. Values are ranges of experimental data obtained for biological duplicates. ND, not detected.

b Calculated with reference to the volume of liquid medium.

c Calculated with the total amount of CO consumed by the end of the incubation time.

d The reported values for acetate as a fermentation product represent the difference between the final and initial acetate concentrations.

e CO could not be quantified in one of the duplicates.

### Effect of initial acetate concentrations on propionate production by cocultures of *Ac. wieringae* strain JM and *An. neopropionicum*.

**FIG 2:**
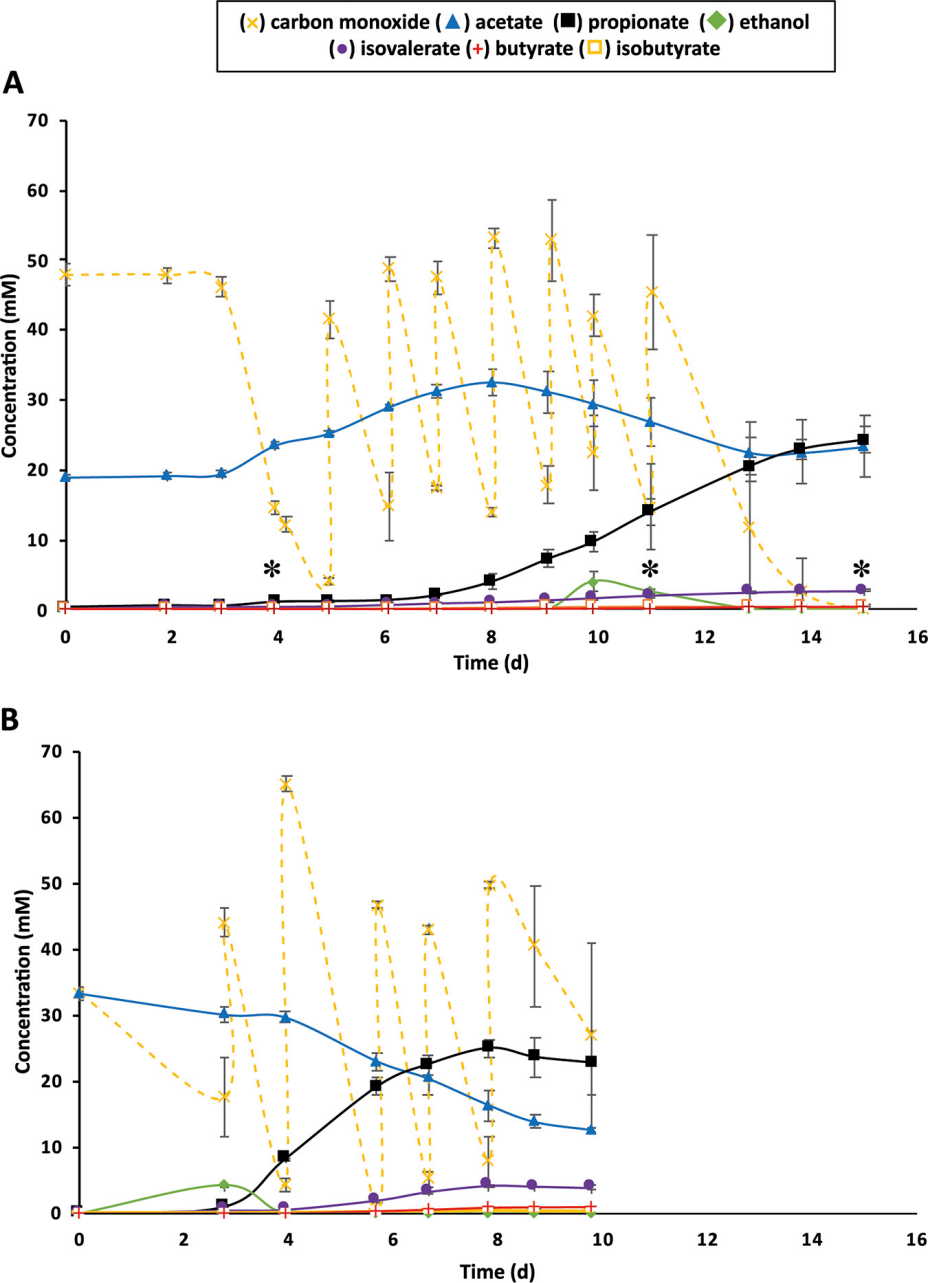
Effect of two different initial acetate concentrations on the growth of the *JM*-*An* coculture under a CO-N_2_-CO_2_ (50%:30%:20% [vol/vol]) headspace with (A) 20 mM acetate or (B) 30 mM acetate. Asterisks indicate sampling time points for proteomic analyses. Error bars represent the standard deviations for biological duplicates (where error bars are not visible, the symbol is greater than the standard deviation).

Conversion of CO to propionate by the coculture *JM*-*An* was studied in relation to the initial acetate concentrations (Fig. 2). Growth of *JM*-*An* on CO with ∼20 mM acetate (Fig. 2A) was characterized by an acetogenic phase during the first 8 days, where the acetate concentration gradually increased to 32.6 mM, followed by acetate uptake and a gradual decrease of the acetate concentration to 22.5 mM (day 13). Upon acetate uptake (days 10 and 11), ethanol was detected (4.0 and 2.5 mM, respectively), and the propionate concentration started to increase. At the end of the experiment (day 15), the product concentrations were 24.3 mM propionate, 2.7 mM isovalerate, 0.5 mM butyrate, and 0.3 mM isobutyrate.

The increment in initial acetate concentration to ∼30 mM resulted in faster propionate production (23 mM in 10 days), higher production of isovalerate (4 mM) and butyrate (0.9 mM), and 0.3 mM isobutyrate (Fig. 2B). Acetate buildup in the medium was not observed; instead, the acetate concentration gradually decreased from 33.3 to 12.7 mM at the end of fermentation (Fig. 2B). In both experiments, acetate supplementation did not seem to affect CO consumption, as CO was consumed regardless of acetate being produced or utilized (Fig. 2). In assays supplemented with 30 mM acetate, ethanol was detected earlier (day 3, 4.1 mM), concurrent with the beginning of propionate production (Fig. 2B).

**FIG 3:**
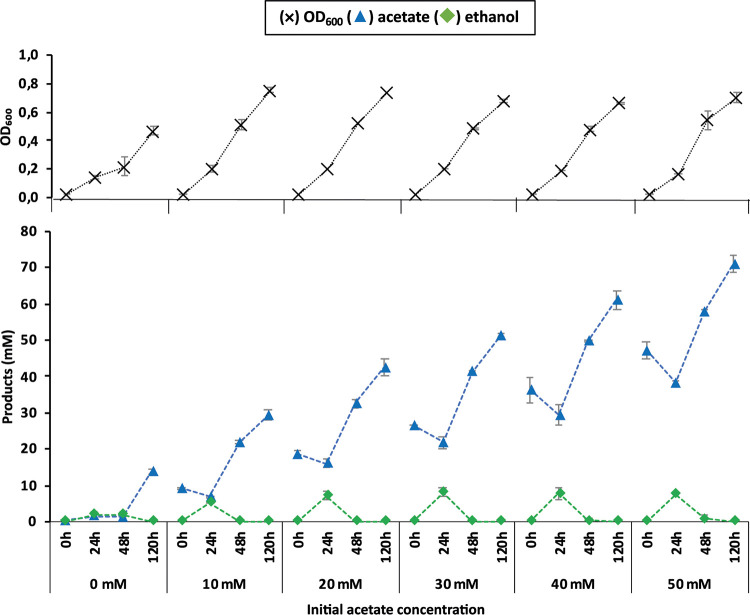
Response of pure cultures of *Ac. wieringae* strain JM to different initial acetate concentrations (0 to 50 mM). Cultures were grown under a CO-N_2_-CO_2_ (50%:30%:20% [vol/vol]) headspace. The optical density and concentrations of acetate and ethanol at 0, 24, 48, and 120 h of growth are displayed. Error bars represent the standard deviations for biological duplicates (where error bars are not visible, the symbol is greater than the standard deviation).

We hypothesized that the switch from the acetogenic to the solventogenic phase in strain JM was induced by the initial high acetate concentrations, which would promote the subsequent conversion to propionate by propionigenic bacteria. To ascertain this, the effect of acetate supplementation on strain JM was also tested (incubations with different initial acetate concentrations and the headspace pressurized with 150 kPa CO-N_2_-CO_2_, 50%:30%:20% [vol/vol]) (Fig. 3). The addition of 10 mM acetate or higher (up to 50 mM) stimulated growth of strain JM. In the absence of acetate at the start of the incubations, strain JM produced low concentrations of ethanol and acetate (1.1 and 1.9 mM, respectively, after 24 h of incubation). However, when acetate was supplemented, ethanol production was promoted and higher concentrations were transiently accumulated in the medium (10.6 to 14.8 mM, after 24 h incubation).

### Proteome analyses of cocultures of *Ac. wieringae* strain JM and *An. neopropionicum*.

Proteome analyses of *JM*-*An* cocultures were conducted at three different stages of cocultivation: after the initial depletion of CO (day 4), during transient ethanol accumulation in the medium (day 11), and at the end of the fermentation (day 15) (Fig. 2A). The aim was to evaluate differences in protein abundance between the acetogenic phase (day 4) and the propionigenic phase (days 11 and 15). A total of 1,624 proteins were detected and quantified: 1,090 were identified as proteins belonging to strain JM and 534 to *An. neopropionicum*. Proteomes from days 11 and 15 were similar. The comparative analysis below concentrates on proteomes from days 4 and 15.

Key enzymes from the Wood-Ljungdahl pathway involved in the conversion of CO to acetyl-CoA were detected in all the analyzed time points, as well as the enzymes involved in acetate and ethanol formation (e.g., acetate kinase, several acetyltransferases, acetaldehyde dehydrogenase, and alcohol dehydrogenases) (Tables S1, S2, and S3). Enzymes of the acrylate pathway in *An. neopropionicum* were also detected in all growth phases, but these were more abundant at the two latter sampling points (Tables S1, S2, and S3). Proteins that changed significantly (*P* < 0.05) and with a log fold change of >1.3 between days 4 and 15 were mainly related to amino acid metabolism, DNA replication, vitamin metabolism, and stress (Tables S1, S2, and S3; Table 2). The proteome of *An. neopropionicum* at day 15 showed a significantly higher abundance of proteins related to the metabolism of tricarboxylic acid (TCA) cycle-derived amino acids (e.g., glutamate) and pyruvate-derived amino acids (e.g., serine); two proteins related to isoleucine metabolism and proline isomerization significantly decreased in abundance (Table 2). Regarding protein abundance in *Ac. wieringae* strain JM, in the propionigenic phase (days 11 and 15), an ABC transporter related to amino acid transport and proteins associated with tRNA loading (excluding methionine tRNA ligase) decreased in abundance. In addition, some proteins related to associated reactions of DNA synthesis and vitamin B_6_ production were less abundant (Table 2).

**TABLE 2:** Proteins of *An. neopropionicum* and *Ac. wieringae* strain JM with significant changes (*P* value of <0.05 and a >1.3 log fold change) between days 4 and 15

Organism	Role	Protein	EC no.^*a*^	Pathway	Log fold change (day 4 vs. day 15)
*An. neopropionicum*	Vitamins	Riboflavin synthase	2.5.1.9	Vitamin B_2_ metabolism	3.2 ↑
		Putative thiamine biosynthesis protein	2.5.1.3	Vitamin B_1_ metabolism	2.1 ↑
		Aminopyrimidine aminohydrolase	3.5.99.2	Vitamin B_1_ metabolism	2.1 ↑
		Riboflavin biosynthesis protein RibBA/D	4.1.99.12	Vitamin B_2_ metabolism	2.4/2.8 ↑
	Amino acids	3-Isopropylmalate dehydratase small subunit	4.2.1.33	Isoleucine metabolism	2.1 ↓
		d-Serine dehydratase	4.3.1.18	Serine metabolism	3.3 ↑
		Aliphatic sulfonates import ATP-binding protein SsuB	7.6.2.14	Sulfated amino acid import	3 ↑
		Methionine import protein	7.4.2.11	Amino acid transport	1.3 ↑
		Glutamate synthase	1.4.1.13	Amino acid synthesis	3.5 ↑
		Aspartate semialdehyde dehydrogenase	1.2.1.11	Amino acid synthesis	3.3 ↑
		Asparagine tRNA ligase	6.1.1.22	Protein assembly	3.6 ↑
		Peptidyl-prolyl *cis*-*trans* isomerase	5.2.1.8	Proline isomerization	2.8 ↓
	Stress	Nitric oxide reductase	1.7.5.2	Redox stress	1.3 ↑
		Sporulation and spore germination	NA	Sporulation	2.6 ↑

*Ac. wieringae* strain JM	Vitamins	Pyridoxamine 5-phosphate oxidase	1.4.3.5	Vitamin B_6_	3.5 ↓
	DNA	DNA topoisomerase 1	5.6.2.1	DNA synthesis	3.3 ↓
		Anaerobic ribonucleoside-triphosphate reductase	1.17.4.2	DNA synthesis	3.1 ↓
		Dihydroorotate dehydrogenase	1.3.3.1	Pyrimidine metabolism	3.0 ↓
	Amino acids	Amino acid ABC transporter	NA	Amino acid transport	2.9 ↓
		Methionyl-tRNA formyltransferase	2.1.2.9	Protein assembly	2.9 ↓
		Methionine tRNA ligase	6.1.1.10	Protein assembly	3.3 ↑
		Glutamate tRNA ligase	6.1.1.17	Protein assembly	3.0 ↓
	Stress	MarR regulator	NA	Antibiotic resistance/oxidative stress	1.8 ↓

a NA, not available in the database.

**FIG 4:**
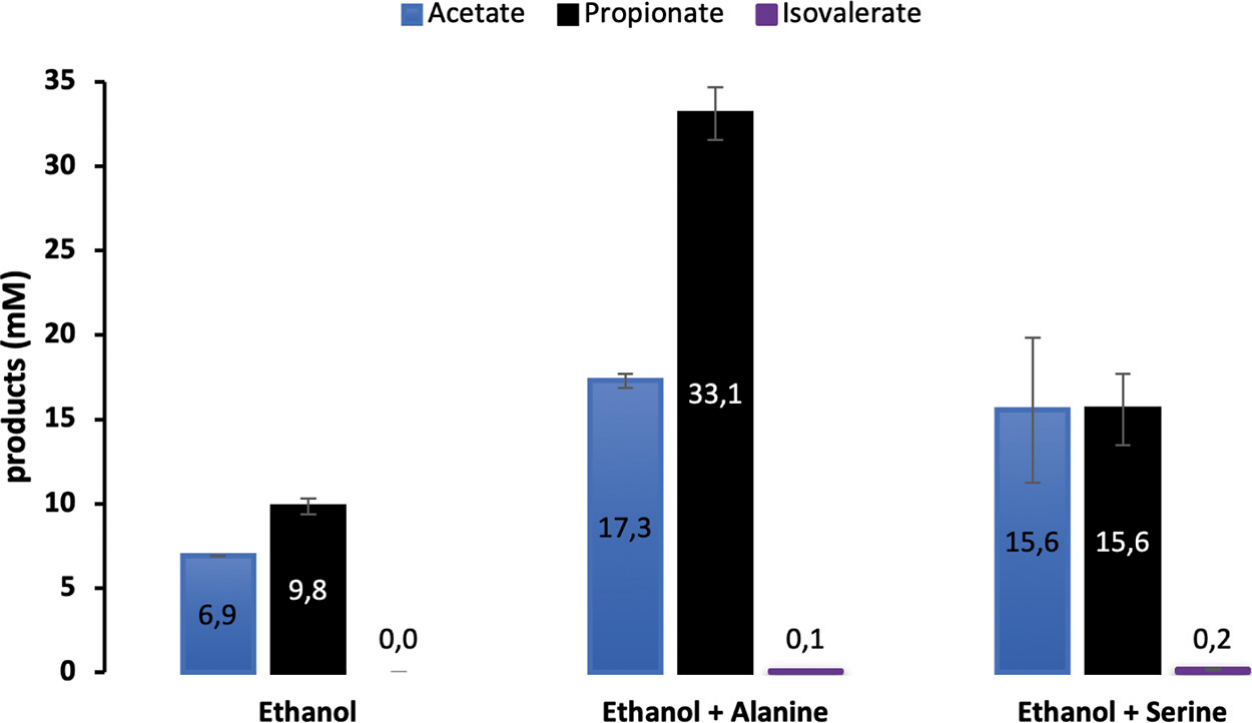
Production profile after the conversion of either ethanol, ethanol plus alanine, or ethanol plus serine by pure cultures of *An. neopropionicum* under a N_2_-CO_2_ (80%:20% [vol/vol]) headspace. Error bars represent standard deviations for biological duplicates.

Because changes in abundance of proteins related to the amino acid metabolism were observed concomitantly for strain JM and *An. neopropionicum*, we speculate that the two bacteria may engage in amino acid interchange. Utilization of amino acids (serine and alanine) by *An. neopropionicum* was tested, in the presence of ethanol. Growth was observed both with ethanol (20 mM) and serine (20 mM) and with ethanol and alanine (20 mM). Ethanol and the respective amino acid were consumed simultaneously over the course of 3 to 5 days. The main products were acetate and propionate, but small amounts of isovalerate (0.1 to 0.2 mM) were produced in the tests with amino acids (Fig. 4). Butyrate and isobutyrate were not found in these experiments.

## DISCUSSION

**FIG 5:**
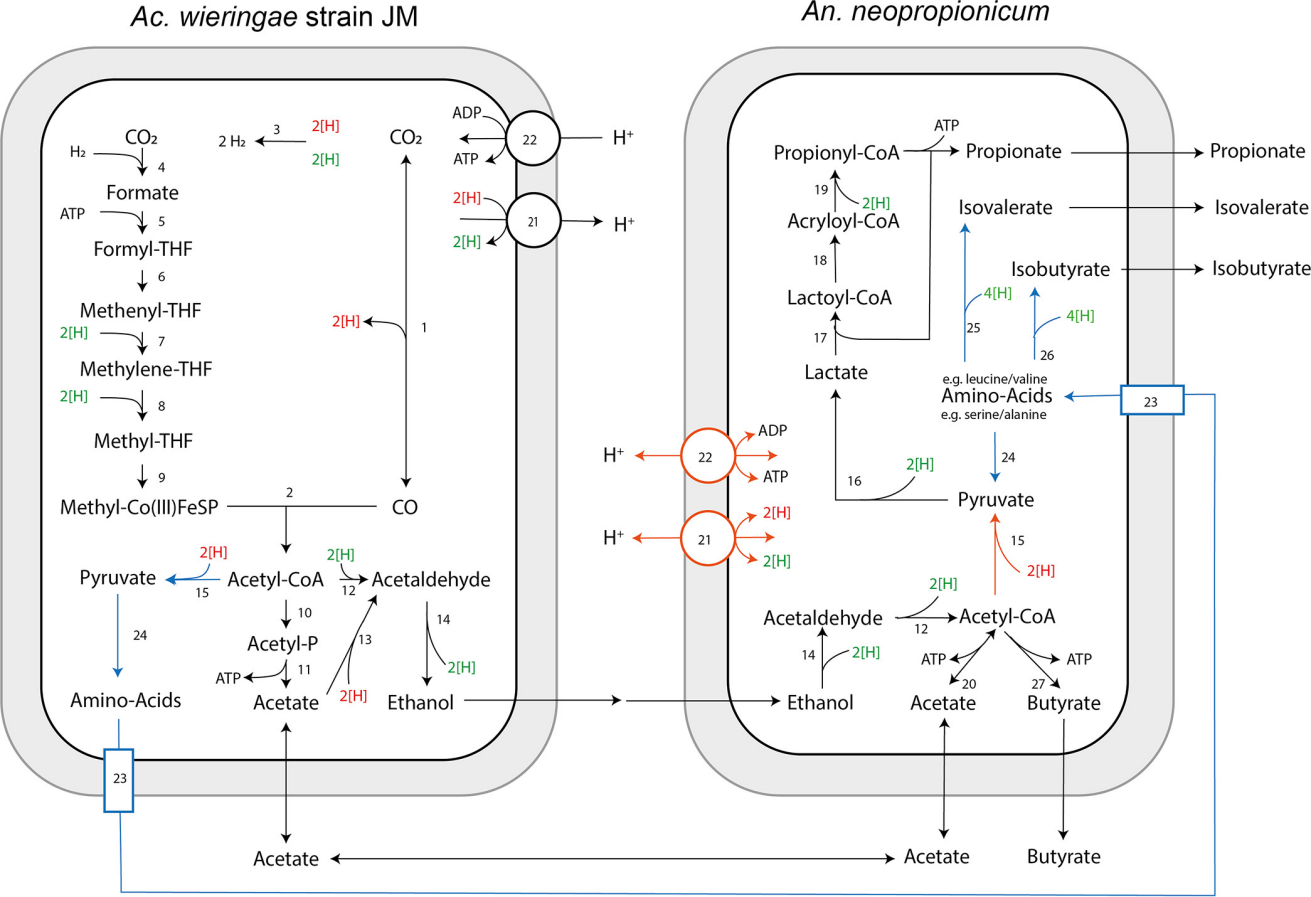
Proposed pathway for propionate production from carbon monoxide by the coculture of *Ac. wieringae* strain JM plus *An. neopropionicum* and their potential amino acid exchange interactions. Reactions in black represent ethanol and acetate exchange and conversion pathways. Reactions in blue represent the amino acid transfer and conversion pathways. Reduction equivalents in red are transferred to ferredoxin, while green equivalents represent NADH/NADPH. The enzymes for the reactions shown are as follows: 1, CO dehydrogenase (CODH); 2, complex CO dehydrogenase/acetyl-CoA synthase (CODH/ACS); 3, hydrogenase; 4, formate hydrogen lyase; 5, formyl-THF synthase; 6, formyl-THF cyclohydrolase; 7, methylene-THF dehydrogenase; 8, methylene-THF reductase; 9, methyltransferase; 10, phosphotransacetylase; 11, acetate kinase; 12, aldehyde-alcohol dehydrogenase; 13, aldehyde ferredoxin oxidoreductase (AOR); 14, alcohol dehydrogenase; 15, pyruvate synthase; 16, d,l-lactate dehydrogenases and lactate racemase; 17, propionate-CoA transferase; 18, lactoyl-CoA dehydratase; 19, acyl-CoA dehydrogenase; 20, acetyl-CoA synthetase; 21, RnF complex; 22, ATPase complex; 23, amino acid transporter; 24, amino acid production/consumption pathways; 25 and 26, multiple-step reactions for isovalerate/isobutyrate biosynthesis from branched-chain amino acids (e.g., leucine and isoleucine); 27, multiple-step reaction for butyrate production. THF, tetrahydrofolate.

The cocultivation of acetogenic bacteria (*Ac. wieringae* type strain or *Ac. wieringae* strain JM) with propionigenic bacteria (*An. neopropionicum* or *P. propionicus*) resulted in the production of propionate from CO. This is a demonstration on how C_1_ compound-fixing and C_3_ compound-producing microorganisms can be combined to expand the product spectrum of syngas fermentation. *Ac. wieringae* strain JM utilizes the Wood-Ljungdahl pathway to convert CO to acetate and ethanol, while *An. neopropionicum* can use ethanol to produce propionate and acetate (Fig. 5; equation 5 in Table 3) and can use ethanol and acetate to produce propionate and butyrate (Fig. 5; equations 6 to 8 in Table 3). Equations and energetics of these and other possible conversions are included in Table 3.

**TABLE 3:** Stoichiometry and Gibbs free energy change of the reactions involved in the coculture of *Ac. wieringae* strain JM and *An. neopropionicum* metabolism on CO and acetate

Organism	Reaction	Value of Gibbs free energy change (kJ)^*a*^	Equation
*Ac. wieringae* strain JM	4H_2_ + 2CO_2_ → acetate^−^ + H^+^ + 2H_2_O	−95	1
	4CO + 2H_2_O → acetate^−^ + H^+^ + 2CO_2_	−174	2
	6CO + 3H_2_O → ethanol + 4CO_2_	−224	3
	Acetate^−^ + H^+^ + 2H_2_ → ethanol + H_2_O	−9.6	4

*An. neopropionicum*	3 ethanol + 2HCO_3_^−^ → 2 propionate^−^ + acetate^−^ + H^+^ + 3H_2_O	−124	5
	3H_2_ (6H) + acetate^−^ + H^+^ + HCO_3_^−^ → propionate^−^ + 3H_2_O	−76	6
	Ethanol + acetate^−^ → butyrate^−^ + H_2_O	−39	7
	2H_2_ (4H) + 2 acetate^−^ + H^+^ → butyrate^−^ + 2H_2_O	−48	8
	10H_2_ (20H) + 4HCO_3_^−^ + 3H^+^ → butyrate^−^ + 10H_2_O	−257	9
	Leucine + 3H_2_O → isovalerate^−^ + HCO_3_^−^ + H^+^ + NH_4_^+^ + 2H_2_	+4.2	10
	5 leucine + 5H_2_O → 5 isovalerate^−^ + butyrate^−^ + 5NH_4_^+^ + HCO_3_^−^ + 2H^+^	−236	11
	Valine + 3H_2_O → isobutyrate^−^ + HCO_3_^−^ + H^+^ + NH_4_^+^ + 2H_2_	+9.7	12

a Calculated from references 21 and 22.

A maximum propionate titer of 24 mM from CO fermentation was obtained during batch cocultivation of strain JM and *An. neopropionicum* (Fig. 2A). This work constitutes a first proof of concept, and product yield can potentially be improved by continuous cultivation and process optimization. Propionate production by the coculture is highly dependent on the ethanol production by the acetogenic strain. Some studies have shown that ethanol productivity can be increased in *Clostridium* strains by process optimization (23, 24). Ethanol production in acetogens can occur via acetyl-CoA, which is reduced in two subsequent reactions catalyzed by an alcohol dehydrogenase (ADH) (first to acetaldehyde and, subsequently, to ethanol) (23). In addition, acetogens can also take up acetate and reduce it to acetaldehyde via a ferredoxin-dependent aldehyde oxidoreductase (AOR) and further to ethanol via ADH (25). AOR was expressed by *Ac. wieringae* strain JM, along with one ADH complex containing a type 2 alcohol dehydrogenase (Tables S1, S2 and S3). This indicates the ability of strain JM to produce ethanol directly via acetyl-CoA and indirectly via acetate.

The oxidation of CO by the CODH complex reduces ferredoxin, providing electrons for the AOR reaction (26). However, acetate is preferably produced by acetogens because it generates one ATP molecule through the enzyme acetate kinase (substrate-level phosphorylation), which does not happen when acetyl-CoA is directly reduced to ethanol (27). Transition from the acetogenic to the solventogenic phase by *Ac. wieringae* strain JM is crucial for the growth of *An. neopropionicum*, which requires ethanol to produce propionate. It was shown previously that acetate addition stimulates ethanol production via the indirect route (via AOR) (28). In addition, a previous study indicated that acetogenic/solventogenic metabolism is controlled by thermodynamics in gas-fermenting acetogens, and cocultivation with ethanol-utilizing microorganisms (because it keeps the ethanol concentration in the medium low) promotes electron flux in acetogens toward ethanol production (28). In the present study, comparison of the proteomes from days 4 and 15 shows no significant changes in abundance of proteins related with the central carbon and energy metabolism in the acetogen (Tables S1, S2, and S3; Table 2). During cocultivation of *JM*-*An*, high concentrations of acetate (∼30 mM) seemed to be associated with the start of the solventogenic phase by strain JM, as ethanol was detected when acetate increased to about 30 mM (Fig. 2A, 8-day time point). Additionally, upon addition of ∼30 mM acetate, immediate ethanol and propionate production was observed (Fig. 2B). When strain JM was cultivated in pure culture, the addition of acetate to the medium led to transient ethanol accumulation (Fig. 3). In the coculture *JM*-*An*, the continuous ethanol removal from the medium by *An. neopropionicum* kept the ethanol concentration low (favoring thermodynamics of ethanol production by the acetogenic strain).

The proteome of *An. neopropionicum* in the coculture *JM*-*An* showed the presence of the enzymes acetate kinase and acetate CoA-transferase (Tables S1, S2, and S3), confirming the ability of *An. neopropionicum* to produce/consume acetate. Ethanol fermentation to propionate (equation 5 in Table 3) is more exergonic than acetate fermentation to propionate (equation 6 in Table 3) and does not require H_2_. Experimentally, it was observed that *An. neopropionicum* cannot grow on acetate with H_2_ (equation 6 in Table 3) (19), but it can use acetate with ethanol to produce butyrate (equation 7 in Table 3) (19), and it can also use acetate in the presence of leucine and/or valine to produce propionate, isovalerate, and/or isobutyrate (equations 6 and 10 to 12 in Table 3) (29). In the coculture *JM*-*An*, both microorganisms can produce or use acetate under certain conditions; therefore, the mechanisms responsible for the net production of ∼4 mM acetate (Fig. 2A) from CO are still unclear.

All the enzymes for operating the acrylate pathway and converting ethanol to propionate could be detected in the proteome of *An. neopropionicum* (Fig. 5; Tables S1, S2, and S3). Two alcohol dehydrogenases were detected in *An. neopropionicum*; one of them is a NADP-dependent dehydrogenase catalyzing the oxidation of ethanol to acetaldehyde (19). Aldehyde-alcohol dehydrogenase, pyruvate synthase, d- or l-lactate dehydrogenase, and lactate racemase perform the subsequent reactions, leading to the formation of lactate, which then goes through the acrylate cycle to yield propionate. Although Tholozan et al. suggest a propionyl-CoA dehydrogenase to catalyze the formation of propionyl-CoA from acrylyl-CoA (19), our proteome analysis indicates the presence of an acyl-CoA dehydrogenase (Tables S1, S2, and S3), as was also proposed by Papoutsakis and Meyer (30).

A striking change in the proteome of *JM*-*An* during cocultivation is related to amino acid metabolism and amino acid transport in both bacterial strains (Table 2). From the literature, it is known that *An. neopropionicum* is capable of utilizing several amino acids (including serine and alanine) as the substrate (19) and that the utilization of branched-chained amino acids stimulates the production of branched-chained fatty acids (31). For example, isovalerate was produced by *An. neopropionicum* from l-leucine (31) but not from ethanol (29). From the literature, isovalerate production from l-leucine was increased in the presence of acetate (29). Here, we show that *An. neopropionicum* could utilize alanine or serine simultaneously with ethanol, generating propionate, acetate, and isovalerate as end products; isovalerate was produced only in the presence of amino acids (Fig. 4). Theoretically, the oxidation of branched-chain amino acids such as leucine to isovalerate is endergonic under standard conditions (equation 10 in Table 3), but the removal of reducing equivalents could render the reaction thermodynamically possible. Similar to *Clostridium* species, *An. neopropionicum* may utilize branched-chain amino acids via the so-called Stickland pathway, ultimately leading to the formation of ATP (32–34).

H_2_ production was not detected in the cocultures of *JM*-*An*, but as *Ac. wieringae* strain JM can utilize H_2_ (equation 1 in Table 3), it was not possible to assess if H_2_ was yielded from amino acid conversion in the coculture. In addition, *An. neopropionicum* could also be using acetate as electron sink during amino acid catabolism, resulting in propionate production (equation 6 in Table 3) (29). We hypothesize that amino acid transfer interaction between *Ac. wieringae* strain JM and *An. neopropionicum* took place, since isovalerate and isobutyrate were produced by *JM*-*An* cocultures (Fig. 2; Table 1). This interaction would require *Ac. wieringae* strain JM to produce leucine and valine, yet proteomics results were not clear on which amino acids could be produced/transferred. Previous research showed that production of amino acids such as alanine by species of the genus *Acetobacterium* is possible (35).

In addition to transfer of carbon/energy from one partner to another, exchange of an amino acid metabolically close to pyruvate (e.g., alanine or serine) could potentially save energy for *An. neopropionicum* by employing pyruvate:ferredoxin oxidoreductase (PFOR), which catalyzes the oxidative decarboxylation of pyruvate to acetyl-CoA and CO_2_, with ferredoxin serving as the electron acceptor (36). This would require less ferredoxin to be generated via the ferredoxin:NAD^+^ oxidoreductase (Rnf), resulting in potential energy conservation. The proteomes of both strains showed signs of stress, including less abundance of DNA replication proteins and more abundance of oxidative stress, sporulation, and antibiotic resistance proteins (Table 2). It could be that the uptake of amino acids by *An. neopropionicum* might have a negative influence on strain JM, forcing it to alter assimilatory pathways to balance its metabolism. More detailed studies under different growth conditions are necessary to assess the theory of amino acid transfer between *Ac. wieringae* strain JM and *An. neopropionicum.*

### Conclusions.

The synthetic coculture composed of *Ac. wieringae* strain JM and *An. neopropionicum* was capable of converting CO to propionate. The transition from acetogenic to solventogenic growth by strain JM is a crucial step, since ethanol is the key intermediate for propionate production by *An. neopropionicum*. The transition from the acetogenic to solventogenic phase in strain JM is correlated with acetate concentrations in the medium.

Proteome analyses of the coculture grown on CO-acetate indicate that the Wood-Ljungdahl and acrylate pathways are used for propionate production, with ethanol and potentially also amino acids being exchanged between the two microbes. Amino acid exchange could be the reason for the production of isovalerate, although this needs to be further assessed. It does highlight the fact that microbial interactions, even in simple systems composed of two microbes, are far more complex than generally considered.

Overall, the coculture of *Ac. wieringae* strain JM and *An. neopropionicum* is another example of how the production spectrum of CO fermentation can be expanded using mixed microbial cultures.

## MATERIALS AND METHODS

### Microorganisms and cultivation.

Acetobacterium wieringae (DSM 1911^T^), Pelobacter propionicus (DSM 2379^T^), and Anaerotignum neopropionicum (DSM 3847^T^) were purchased from DSMZ (German Collection of Microorganisms and Cell Cultures, Braunschweig, Germany). Acetobacterium wieringae strain JM was obtained from our own culture collection (15). Freeze-dried cultures of *Ac. wieringae*, *P. propionicus*, and *An. neopropionicum* were activated using the recommended media DSM-135, DSM-298, and DSM-318b, respectively. Further cultivation of these strains and cocultivation assays were done in anaerobic basal medium prepared as described previously (37), with the addition of yeast extract (0.1 g liter^−1^), formate (1 mM), and phosphate buffer, pH 7.0 (K_2_HPO_4_/KH_2_PO_4_, 10 mM). The headspace was filled with the desired gas (i.e., CO-CO_2_-N_2_, 50%:20%:30% [vol/vol], or CO_2_-N_2_, 20%:80% [vol/vol]) to a final pressure of 170 kPa. Bottles with medium were autoclaved at 120°C for 20 min. Before inoculation, the medium was supplemented with vitamins and reduced with ∼0.8 mM sodium sulfide (Na_2_S·9H_2_O) from an anaerobic sterile stock solution (30). The final pH of the medium was 7.0 to 7.2. Sodium acetate was added from a 1 M anaerobic and sterile stock solution.

### Pure-culture experiments.

The acetogenic bacteria *Ac. wieringae* type strain and *Ac. wieringae* strain JM were grown in the media described above, with the addition of 1 mM formate and 0.1 g liter^−1^ yeast extract, under the headspace of CO, N_2_ and CO_2_ (50%:30%:20%, vol/vol), at 30°C with 130 rpm shaking. The propionigenic bacteria *P. propionicus* and *An. neopropionicum* were grown in the media described above with 20 mM ethanol, supplemented with 1 mM formate and 0.1 g liter^−1^ yeast extract, under the headspace of N_2_ and CO_2_ (80%:20%, vol/vol), at 30°C under nonshaking conditions. To test the effect of acetate in the acidogenic/solventogenic metabolism of *Ac. wieringae* strain JM, pure cultures of this organism were grown in the media described above for acetogens, in duplicates for the concentrations of 0, 10, 20, 30, 40, and 50 mM acetate. To test if *An. neopropionicum* can simultaneously use ethanol and pyruvate-derived amino acids, incubations were made in medium as described above for propionigenic bacteria, in duplicates containing either 20 mM ethanol or 20 mM ethanol plus 20 mM either l-serine or l-alanine as the substrate.

### Coculture experiments.

The following four cocultures were constructed: *Ac. wieringae* plus *P. propionicus* (*Aw*-*Pp*), *Ac. wieringae* plus *An. neopropionicum* (*Aw*-*An*), *Ac. wieringae* strain JM plus *P. propionicus* (*JM*-*Pp*), and *Ac. wieringae* strain JM plus *An. neopropionicum* (*JM*-*An*). After growing until late exponential phase, pure cultures of the different organisms were combined in a ratio of 1:1 (culture volume), initiating the cocultivation. The headspace was then N_2_ washed and repressurized with 170 kPa of CO, N_2_, and CO_2_ (50%:30%:20%, vol/vol). The bottles were further incubated. For further experiments with cocultures, a 2% inoculum of previously grown coculture was used. The culturing conditions were as described above for acetogenic bacteria. In assays requiring subsequent CO additions, the headspace was refilled using a sterile 0.22-μm filter to keep the gas flow aseptic.

### Analytical techniques.

Organic acids and alcohols were analyzed via high-performance liquid chromatography (HPLC) on a system equipped with a MetaCarb 67H column (Agilent Technologies, Santa Clara, CA). The column was operated at a temperature of 45°C with a flow rate of 0.8 ml/min. Detection was done via a refractive index (RI) and UV detector. H_2_SO_4_ (0.01 N) was used as the eluent. Samples of 1.0 ml were taken and immediately centrifuged at 13,000 × *g*. Subsequently, vials for HPLC analysis were prepared with the supernatant and 30 mM arabinose solution as the internal standard at a ratio of 8:2 (vol/vol). Gas analysis was done by gas chromatography (GC). Gas samples of 0.2 ml were taken using a 1-ml syringe and analyzed in a Compact GC 4.0 (Global Analyser Solutions, Breda, The Netherlands). CO and H_2_ were measured using a Molsieve 5A column operated at 100°C, coupled to a Carboxen 1010 precolumn. CO_2_ was measured using an Rt-Q-Bond column operated at 80°C. Detection was done via a thermal conductivity detector.

### Sample preparation for proteomics.

The cocultures of *Ac. wieringae* strain JM and *An. neopropionicum* were grown in nine 0.5-liter bottles, filled with 0.2 liter of basal medium under the culturing conditions described above for acetogenic bacteria, except that in the first 3 days of growth, nonshaking conditions were used. Cultures were sampled at the 4th, 11th, and 15th days of incubation, by three bottles at each selected time point. Before cell harvesting by centrifugation (10 min, 4°C, 16,000 × *g*), cultures were quickly cooled on iced water for 20 min to decrease cell activity. Cell pellets were resuspended in 0.5 ml SDS lysis buffer (100 mM Tris-HCl, pH 7.5; 4% SDS [wt/vol]) plus 50 μl 1 mM PMSF (phenylmethylsulfonyl fluoride) and sonicated six times (30-s pulse, 30-s rest) on ice. Cell debris was removed by centrifugation (13,000 × *g*, 10 min). The final protein concentration, for LC-MS/MS analysis, was assessed using the Pierce bicinchoninic acid (BCA) protein assay kit (Life Technologies, Thermo Fisher Scientific, Waltham, MA), following the manufacturer’s instructions.

Samples were further subjected to a short protein separation (ca. 4 cm) using 12% Mini-Protean TGX precast protein gels (12 well, 20 μl; Bio-Rad, Hercules, CA), loading approximately 60 μg proteins per well. Reduction of cysteine disulfide bridges occurred from the addition of 50 mM NH_4_HCO_3_ plus 10 mM dithiothreitol, pH 8, and overnight incubation at room temperature. Reforming of disulfide bridges was inhibited via alkylation of reduced cysteines by adding 100 mM Tris (pH 8) plus 20 mM iodoacetamide (pH 8) and subsequently incubating at room temperature in the dark, with slow shaking for 1 h. Afterwards, the gel lane for each sample was cut into four slices, proteins were digested by adding 100 μl of a 5 ng μl^−1^ trypsin solution prepared in 1.5 mM ammonium bicarbonate and subsequently incubating overnight at room temperature. The extraction of peptides was performed by adding 10% trifluoroacetic acid with a pH between 2.0 and 4.0. Samples were further cleaned up with C_18_ microcolumns. The microcolumns were prepared with two C_18_ Empore disks transferred to 200-μl tips, with 4 μl of a 50% slurry of Lichroprep C_18_ material in methanol, and further washed with 200 μl methanol and equilibrated with 100 μl of a 0.1% (vol/vol) formic acid solution. The samples were transferred to the microcolumns and washed with 200 μl of a 0.1% (vol/vol) formic acid solution. Peptides were eluted with 50 μl 50% acetonitrile and 50% of 0.1% (vol/vol) formic acid. The acetonitrile content was reduced by putting the samples in a concentrator at 45°C for 2 h. The final volume for LC-MS analysis was 20 μl.

### LC-MS data acquisition.

Peptides from the protein samples obtained from the three biological triplicates were analyzed by injecting 18 μl sample over a 0.10- by 32-mm Magic C_18_ AQ 200A preconcentration column (prepared in-house) with 5-μm beads (Bruker Nederland B.V.) at a constant pressure of 800 bar (normally resulting in a flow of ca. 7 μl/min). Peptides were eluted from the preconcentration column onto a 0.10- by 250-mm ReproSil-Pur 120 C_18_ AQ analytical column (prepared in-house) with 1.9-μm beads with an acetonitrile gradient at a flow rate of 0.5 μl/min with a Proxeon Easy nanoLC II. The gradient consisted of an increase from 9% to 34% acetonitrile in water with 0.1% (vol/vol) formic acid solution for 50 min followed by a fast increase in the percentage acetonitrile to 80% (with 20% water and 0.5% [vol/vol] acetic acid–0.1% [vol/vol] formic acid in both the acetonitrile and the water) for 3 min as a column-cleaning step.

An electrospray potential of 3.5 kV was applied directly to the eluent via a stainless-steel needle fitted into the waste line of the microcross that was connected between the preconcentration column and the analytical column. Full-scan positive-mode Fourier transform MS (FTMS) spectra were measured between *m/z* 380 and 1,400 on an LTQ-Orbitrap XL in the Orbitrap at high resolution (60,000). Ion trap (IT) and Orbitrap automatic gain control (AGC) targets were set to 10,000 and 500,000, respectively, or maximum ion injection times of 100 μs and 500 ms. Collision-induced dissociation (CID) fragmented (isolation width, 2 *m/z*; 30% normalized collision energy) MS/MS scans of the four most abundant 2 and 3+ charged peaks in the FTMS scan were recorded in data-dependent mode in the linear trap (MS/MS threshold = 5.000; 45-s exclusion duration for the selected *m/z* ± 25 ppm).

### Proteome analyses.

Protein identification and relative quantitation were performed with MaxQuant software (v.1.6.3.4) (38) in the “Specific Trypsin/P” digestion mode with a maximum of two missed cleavages. Extracted MS/MS spectra were searched against *Ac. wieringae* strain JM and *An. neopropionicum* protein sequence databases downloaded from NCBI UniProt. Amino acid sequences of known contaminant proteins (e.g., skin and hair proteins, trypsin, and LysC) were used as the contaminants database. The following settings were used for peptide and protein identification: carbamidomethyl (Cys) as a fixed modification; acetyl (protein N-term), oxidation (M), and deamidation (NQ) as variable modifications; predefined MS (Orbitrap) and MS/MS (ion trap) settings; a minimal peptide length of seven amino acids; and a maximum allowed false discovery rate of 1% at both the peptide and protein levels. Label-free quantitation (LFQ) was performed with the match between runs and requantify options on using at least two peptides, at least one of which is unique. Retention time alignment was performed with a time alignment window of 20 min and a retention time match window of 0.7 min. LFQ values were used for subsequent data analysis. Reversed hits were deleted from the MaxQuant result table as well as all results showing a normalized label-free quantitation intensity value of 0 for both sample and control. The normal logarithm was taken from protein LFQ MS1 intensities as obtained from MaxQuant. Log LFQ values of 0 were replaced by a value of 4.6 (a value slightly lower than the lowest measured value) to make sensible ratio calculations possible. Relative protein quantitation of sample to control was done with Perseus (39) by applying a two-sample *t* test using the LFQ intensity columns obtained. Nano-LC-MS/MS system quality was checked with PTXQC (40) using the MaxQuant result files.

### Data availability.

The data sets supporting the conclusions of this article are included within the article and in Fig. S1 and Tables S1, S2, and S3. The mass spectrometry proteomics data have been deposited in the ProteomeXchange Consortium via the PRIDE (41) partner repository with the data set identifier PXD020960.
