# Supplementary material for: Propionate Production from Carbon Monoxide by Synthetic Cocultures of Acetobacterium wieringae and Propionigenic Bacteria
Source: Appl Environ Microbiol. 2021 Jun 25;87(14):e02839-20. doi: 10.1128/AEM.02839-20 (PMC8231444; doi:10.1128/AEM.02839-20)
Supplement: Supplemental file 1 — Figure S1. Download AEM02839-20_Supp_1_seq3.pdf, PDF file, 0.2 MB [file aem02839-20_supp_1_seq3.pdf]

Supplementary information for

Propionate production from carbon monoxide by synthetic co-cultures of *Acetobacterium wieringae* spp. and propionigenic bacteria

João P. C. Moreira[a,b], Martijn Diender[b], Ana L. Arantes[a], Sjef Boeren[c], Alfons J.M. Stams[a,b], M. Madalena Alves[a], Joana I. Alves[a], Diana Z. Sousa[b*]

[a] Centre of Biological Engineering, University of Minho, 4710-057 Braga, Portugal

[b] Laboratory of Microbiology, Wageningen University & Research, Stippeneng 4, 6708 WE Wageningen, The Netherlands

[c] Laboratory of Biochemistry, Wageningen University & Research, Stippeneng 4, 6708 WE Wageningen, The Netherlands

*Corresponding author: Diana Z. Sousa

Tel: +31317483107

Email: diana.sousa@wur.nl

Running title: Microbial propionate production from CO

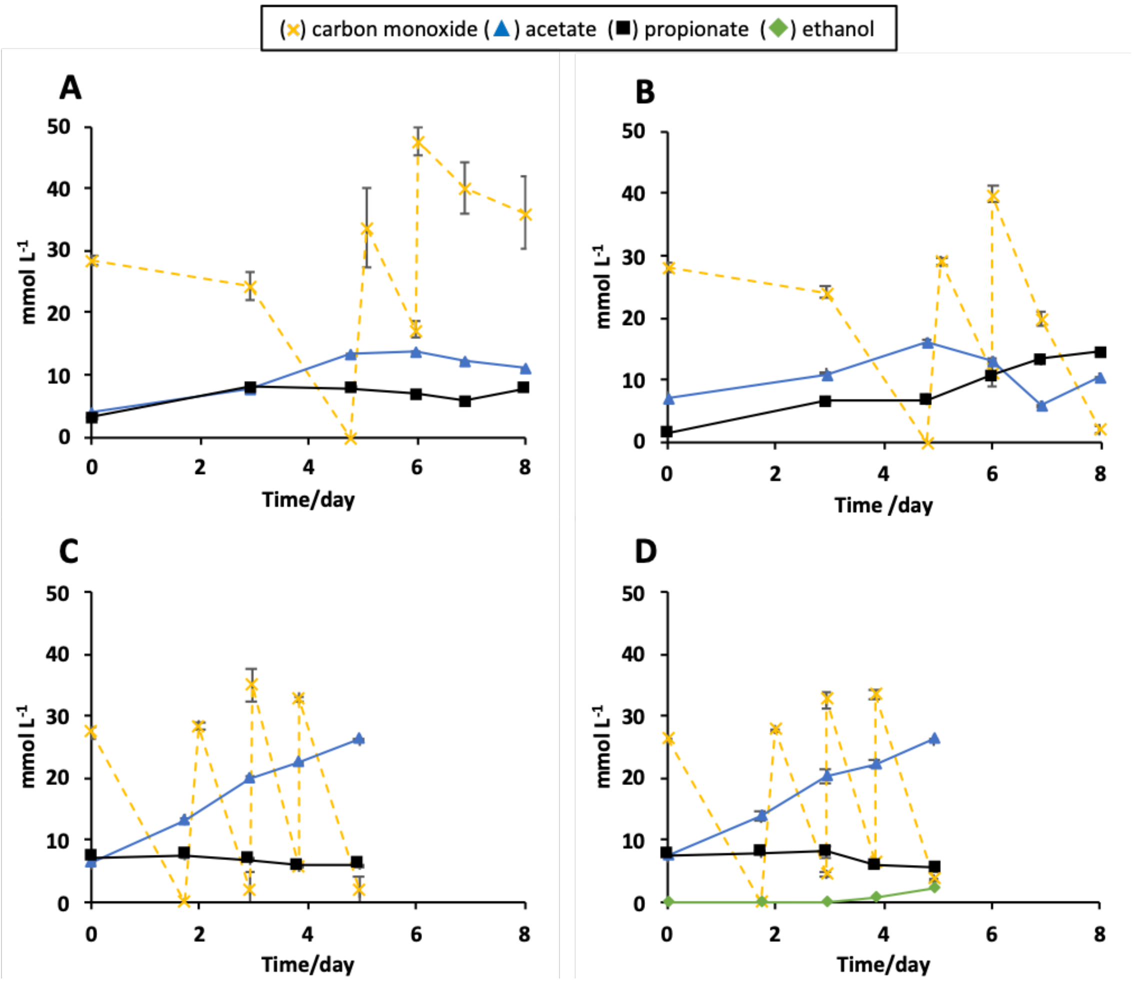

Fig. S1. Conversion of CO by co-cultures upon fusion of pure cultures at exponential growth. **(a)***Aw-An*; **(b)** *Aw-Pp*; **(c)** *JM-An*; **(d)** *JM*-Pp.
